# Genome-Wide Expression Profiling and Networking Reveals an Imperative Role of IMF-Associated Novel CircRNAs as ceRNA in Pigs

**DOI:** 10.3390/cells11172638

**Published:** 2022-08-24

**Authors:** Salsabeel Yousuf, Ai Li, Hui Feng, Tianyi Lui, Wanlong Huang, Xiuxiu Zhang, Lingli Xie, Xiangyang Miao

**Affiliations:** State Key Laboratory of Animal Nutrition, Institute of Animal Sciences, Chinese Academy of Agricultural Sciences, Beijing 100193, China

**Keywords:** intramuscular adipose tissue, metabolic syndromes, transcriptome analysis, expression profiling, protein modeling, genomic organizations

## Abstract

Intramuscular fat (IMF) deposition is a biological process that has a strong impact on the nutritional and sensorial properties of meat, with relevant consequences on human health. Pork loins determine the effects of marbling on the sensory attributes and meat quality properties, which differ among various pig breeds. This study explores the crosstalk of non-coding RNAs with mRNAs and analyzes the potential pathogenic role of IMF-associated competing endogenous RNA (ceRNA) in IMF tissues, which offer a framework for the functional validation of key/potential genes. A high-throughput whole-genome transcriptome analysis of IMF tissues from longissimus dorsi muscles of Large White (D_JN) and Laiwu (L_JN) pigs resulted in the identification of 283 differentially expressed circRNAs (DECs), including two key circRNAs (circRNA-23437, circRNA-08840) with potential binding sites for multiple miRNAs regulating the whole network. The potential ceRNA mechanism identified the DEC target miRNAs-mRNAs involved in lipid metabolism, fat deposition, meat quality, and metabolic syndrome via the circRNA-miRNA-mRNA network, concluding that ssc-mir-370 is the most important target miRNA shared by both key circRNAs. TGM2, SLC5A6, ECI1, FASN, PER1, SLC25A34, SOD1, and COL5A3 were identified as hub genes through an intensive protein-protein interaction (PPI) network analysis of target genes acquired from the ceRNA regulatory network. Functional enrichments, pathway examinations, and qRT-PCR analyses infer their implications in fat/cholesterol metabolism, insulin secretion, and fatty acid biosynthesis. Here, circRNAs and miRNA sequencing accompanied by computational techniques were performed to analyze their expressions in IMF tissues from the longissimus dorsi muscles of two pig breeds. Their target gene evolutionary trajectories, expression profiling, functional enrichments, subcellular localizations, and structural advances with high-throughput protein modeling, following genomic organizations, will provide new insights into the underlying molecular mechanisms of adipocyte differentiation and IMF deposition and a much-needed qualitative framework for future research to improve meat quality and its role as a biomarker to treat lipid metabolic syndromes.

## 1. Introduction

Intramuscular fat (IMF) is considered an important economic trait associated with meat quality due to its strong impact on the sensory attributes of meat, tenderness, flavor, and water retention capacity; it has relevant consequences on human health [1]. IMF accumulates within/between the muscle fibers; this is known as marbling, which is of great importance in beef and pork loin cuts that increase with the growth and age of animals regulating various genetic mechanisms [2]. Subcutaneous adipose tissue (SCAT) develop under the skin, in the back, and abdomen, with the largest amounts of fat in pork carcasses, adversely affecting the lean meat rate and, hence, is considered waste. IMF content is embedded in body fat [3] and has a significant correlation with metabolic disorders [4], such as insulin resistance, certain tumors, type 2 diabetes, and cardiac diseases [5], as evident in catecholamine-induced lipolysis and insulin-induced lipogenesis [6]. Ayuso, D. et al. [7] reported remarkable differences between the fatty acid profiles of IMF and SCAT in the Iberian pig, which are attributed to their different deposition sites [8] and developments from different cells [9].

Improvements in IMF content via reduced fat quantity in pork are primary concerns for pig farmers because abnormal fat depositions play an important role in the development of obesity and associated disorders [2,10]. However, enhancing IMF content through conventional breeding is complicated and takes time. Recent advances in high-throughput RNA-sequencing have emerged as powerful tools of transcriptome profiling, to explore the genetic architectures of complex traits [11]. It has been revealed in previous studies that the amount of intramuscular fat content in any animal depends on various factors, including genetic makeup, animal breed, nutritional value, weight, age, etc. [12,13,14,15]. The Laiwu pig (L_JN), an exceptional kind of fatty pig from North China, is famous for its excellent meat quality due to its higher water-retaining capability, bright red color, and tenderness [16]; it is considered the best animal model to study the mechanisms of high IMF deposition due to high IMF content (11.6%) [17,18]. Moreover, the Large White pig (D_JN) due to its lower subcutaneous fat and IMF content, is the best-characterized and studied breed of a typical lean pig [19]. 

In large animal models, the desire to better understand the mechanisms of adipose tissue and immunological functions reflects a similar need in humans [5]. Regulatory mechanisms of fat deposition in the SCAT of the longissimus dorsi muscles of two pig breeds have been investigated previously [20], which shows the implication of circRNA target genes in pathways related to fat deposition, adipocyte differentiation, lipid metabolism, and disease-related processes [21]. However, the mechanisms underlying marbling, fat deposition, and the potential pathogenic role of circular RNA as competing endogenous RNA (ceRNA) in pig IMF tissues have not yet been explored. This further signifies the higher differentiation and proliferation rate of adipocytes in the SCAT compared to IMF and the fat-type pig versus the lean type [20,22,23,24]. So, studies on the two kinds of fat cell (IMF and SCAT) metabolism regulations between two pig breeds with opposing features would be worthwhile to explore differences in their genetic makeup, which will be helpful to understand the breeding strategies for regulating the intramuscular and subcutaneous fat depositions of pigs (and in treating associated diseases). 

Some protein-coding genes may produce a class of endogenous non-coding RNA, such as circular RNA, which is more stable and resistant to endonucleases than linear RNA, such as miRNAs [25,26]. Overwhelming evidence has indicated that CircRNAs contribute to the expressions of various genes and the regulatory network of adipogenesis [27,28]. So far, little is known about the individual functions of circRNAs but they have been found expressed in a widespread manner, harboring critical regulatory roles, specifically by sponging miRNAs and RNA-binding proteins. miRNAs, as fine-tuners of post-transcriptional gene regulation, play critical roles in normal development and homeostasis, the dysregulation of which has been implicated in various disorders, such as Alzheimer’s disease [29], cancer [30], cardio-cerebrovascular [10], and neurological disorders [31], as well as in cell growth and development, such as cell proliferation [32], oogenesis [33], and fertilized egg formation [34]. 

With the development of whole-genome sequencing and advanced transcriptome technologies, researchers have uncovered a vast class of ncRNAs in mammalian cells with great potential to regulate gene expression [35,36]. Moreover, regarding the differences in fat accumulation between the two pig breeds, this study aimed to identify DECs and explore their regulatory roles as ceRNA in lipid metabolism through a systematic transcriptome analysis, along with high-throughput protein modeling, co-expression networking, functional enrichments, genomic organizations, and protein-protein interactions. These results may provide therapeutic approaches to treating the anomalies of lipid metabolism. 

## 2. Materials and Methods

### 2.1. Ethics Statement

Experiments and animal care in this study were approved by the “Animal Use and Care committee” of the Animal Sciences Institute, the Chinese Academy of Agricultural Sciences, Beijing China. All experiments were executed under the relevant guidelines and regulations set by the Ministry of Agriculture, the People’s Republic of China.

### 2.2. Experimental Animals and Sample Collection

Large White and Laiwu (local) pig breeds were used as experimental animals due to their differences in fat deposition; they were raised in the same environment at Daqian Agriculture and Animal Husbandry Co., Ltd. (Laiwu City, China). In total, six samples from female pigs of two breeds (three from each breed) were used. They were fed according to their up-to-date nutritional needs (Chinese pig feeding standards NY/T 65-2004)). At slaughter age (about 180 days old), their body weights were similar within species (L_JN 40 KG and D_JN 108KG), and were in good health and physical conditions. There were some differences in the growth and carcass qualities among the different pig breeds. As a result, different pig breeds possessed different slaughter weights at the slaughter age. After slaughtering, IMF tissues were stripped from the longissimus dorsi muscle with sterilized ophthalmic forceps, and a scalpel and interfascicular fats were carefully separated to avoid contamination of muscle fibers. The whole process was performed on ice to minimize the RNA degradation following the method given by (Li and Durbin, 2010). Afterward, the tissues were divided into small pieces with sterilized scissors and immediately transferred into liquid nitrogen using 5 mL of cryovials and then stored at −80 °C until further use.

### 2.3. RNA Isolation and Quality Control

TRIzol reagent (Invitrogen Life Technologies, Carlsbad, CA, USA) was used for RNA extraction from each IMF sample as per the manufacturer’s instruction. The total RNA purity and concentration were measured by using a NanoDrop (ND) 1000 spectrophotometer (Thermo Fisher Scientific, Waltham, MA, USA). RNA integrity was tested by denaturing AGE (agarose gel electrophoresis).

### 2.4. RNA Library Construction

RNA libraries were generated through TruSeq Stranded Total RNA LT Kit with Ribo-Zero TM Gold (RS-122-2301) for Illumina sequencing as per the manufacturer’s protocol. The total RNA was extracted and subjected to the Ribo-Zero rRNA Kit for the removal of rRNA (ribosomal RNA). Linear RNAs were removed with RNase R (Epicenter, Madison, WI, United States); to break down circRNAs into fragments. Random hexanucleotide primers were utilized to create first-strand cDNAs using the circRNA fragments as templates. Then, second-strand cDNAs were generated using a second-strand cDNA synthesis mix following the dUTP mix as a substitute for dTTP. At this point, different adapters were ligated at 5′ and 3′ ends of the second strand. The cDNA strand containing dUTP was digested using the uracil N-glycosylase (UNG) enzyme, preserving only the cDNA strand with various adapters. The recovered cDNA was further purified utilizing a cDNA purification kit. After purification, a poly adenylation poly (A) tail was added to cDNA and a sequencing adaptor was ligated at the repair end. Afterward, size selection and PCR amplification were performed. The Agilent 2100 Bioanalyzer was used to examine the quality of the prepared library. After ensuring the quality, Illumina HiSeq^TM^ 2500 was used for library sequencing.

### 2.5. Identification and Characterization of circRNAs

The NGSQC Toolkit (v2.3.3) was used for adapter trimming and quality control of the sequenced data [37] by removing N-bases and low-quality bases to generate high-quality clean reads for further analysis. Then, the Burrows-Wheeler Alignment Tool (BWA) [38] was used for mapping the clean reads to the reference genome, which resulted in the sequence alignment map (SAM) file. In the next step, the SAM file was used as an input source in CIRI (circRNAs identifier tool) [21,39] to recognize the circRNAs from filtered data and to detect the CIGAR values following the mapping position and strand information in the SAM alignment. CIGAR values reflect junction features in the form of upstream xS/HyM and downstream xMyS/H (x, y denote the mapping (M) number symbol S for soft clipping, and H for hard clipping bases). Multiple filtration strategies, such as paired chiastic clipping signals (PCC signals) and paired-end mapping (PEM), were employed to remove false positives. Afterward, GT-AG splicing signals were determined from the junction reads, and the exon boundary positions were extracted from GTF/GFF annotation files. Finally, full sequences with junction sites were realigned using the DP algorithm (dynamic programming) with reference genome sequences to ensure the consistency of the identified circRNAs.

### 2.6. Identification and Characterization of miRNAs

Initially, the data obtained from Illumina HiSeq sequencing, known as raw reads/data, were run through cutadapt software [40] to remove connector sequences. For quality assurance, sequences less than 15 bp and greater than 41 bp were filtered out and counted their type (unique) and quantity (total no) of small RNA (in total) by using the fastx toolkit (version 0.0.13) following length distribution statistics on miRNA. Generally, the length of small RNA ranges from 18 to 30 bp. The peak of the length distribution can help us determine the type of small RNA, such as miRNA concentrated at 21–22 bp, siRNA at 24 bp, and piRNA at 30 bp. Small RNA sequencing data were then compared with different databases; small RNAs, such as tRNA, snRNA, rRNA. etc., were removed as much as possible. Filtered reads were then compared to the above-mentioned databases with the miRBase database (version 21.0) by using Bowtie software for known miRNA and Mirdeep2 [41] to predict novel (unknown) miRNAs.

The identified known miRNA and the newly predicted miRNA expression statistics were normalized to obtain the expressions in transcript per million (TPM) with the normalization formula [42,43]. Similarly, the fold-change and *p*-value were calculated from the normalized expression.

$$Normalized\;Expression\;=\;\frac{Actual\;miRNA\;count}{Total\;count\;of\;clean\;reads\;}\;\times \;10^{6}$$

### 2.7. Differential Expression Analysis and ceRNA Regulatory Networking

DESeq2 [44] was used for differential expression analyses of circRNAs and miRNAs. An absolute value of the |fold change| ≥ 2 and *p*-value < 0.05 with the Benjamini-Hochberg method corrected *p* ≤ 0.05 and was used to screen the differentially expressed circular RNAs (DECs) and miRNAs (DEmiRNA) from L_JN and D_JN samples. Then, the number of up/downregulated circRNAs and miRNAs were obtained. After identifying DECs and their target miRNAs, an interaction network was constructed between them on 26 March 2022, using Cytoscape software (http://apps.cytoscape.org/apps/networkanalyzer), which provided us with node degree distribution. It is well known that nodes with large numbers of interacting partners (edges) are considered hubs/keys in the scale-free biological network, which results in the identification of key/hub/potential DECs. To further investigate the functions and interactions among key potential circRNAs and their target miRNA and mRNAs, we constructed another regulatory network between ncRNAs and mRNAs through Cytoscape software.

Through the interaction of key circRNA-miRNA, it can be useful to examine the functions and mechanisms of circRNA serving as miRNA sponges. Knowing that circRNA contains multiple MREs, the software Miranda (v3.3a) [45] was used to identify the target miRNAs of key potential circRNAs. Target genes of differentially expressed miRNAs were predicted with miRDB and TargetScan (v3.3a) software [46,47]. The circRNA-miRNA-mRNA pairs were predicted through MiRanda software (score threshold ≥ 160, energy cutoff ≤ −20). 

Based on the ceRNA theory, mutual interactions of circRNA and mRNA with miRNA were considered to build the circRNA-miRNA-mRNA network, which was used as the input file in Cytoscape (v3.8.2 version) to visualize their key/hub circRNAs and miRNAs following mRNAs of the ceRNA regulatory network [48].

### 2.8. Functional Enrichment Analysis of DEC Host and Target Genes

The Gene Ontology (GO) enrichment analysis of all DEC host and target genes was accomplished using the cluster Profiler in the R package [49]. GO terms were retrieved through the Benjamini-Hochberg method; terms under *p*-value (*p* < 0.05) were considered significantly enriched. KEGG Orthology-Based Annotation System (KOBAS) software [50] with default settings and parameters was used to examine the statistical enrichment of DEC host genes in the Kyoto Encyclopedia of Genes and Genomes (KEGG) pathway with a *p*-value (*p* < 0.05) for significantly enriched terms. The calculation formulas are given below:

P = 1 − ∑i=0n−1MiN − Mn − iNn              Enrichment Score = mnMn

Note: N represents the number of circRNAs with GO annotations in all circRNAs; n is the number of circRNAs with GO annotations in the differentially expressed circRNAs; M is the number of circRNAs in all circRNAs with the host genes annotated as specific GO terms; m is the host gene annotated as the number of differentially expressed circRNA for specific GO terms. The biological functions of circRNA host genes can be understood based on the results of the GO analysis combined with biological significance.

### 2.9. Phylogenesis and Genomic Structures of Target Genes

The full-length amino acid sequences, CDS, and genomic sequences of target genes were retrieved from the NCBI database (https://www.ncbi.nlm.nih.gov/) on 11 February 2022. To align protein sequences, the ClustalW tool (version 2.0) was used with default settings. Then, MEGA 7.0 was used to construct the neighbor-joining (NJ) tree with default parameters under 1000 bootstrap replicates [51]. The CDS and genomic sequences were used as input files in the “Gene Structure Display Server (GSDS)” (http://gsds.cbi.pku.edu.cn/) on 18 February 2022 to draw the exon/intron distribution.

### 2.10. Expression Profiling and Subcellular Localization of Target Genes

To analyze the tissue-specific expression profiles of target genes, we used FPKM values of RNA-seq data acquired from the transcriptome analysis of IMF tissues from longissimus dorsi mussels of two pig breeds (L_JN and D_JN). The heat map along with the phylogenetic tree was generated through TBtools software [52] for the differential expression analysis. Subcellular localization prediction of IMF tissue-associated target genes of two key circRNA-binding sites was carried out by using several websites, online servers, and tools, such as the TargetP online web server (http://www.cbs.dtu.dk/services/TargetP/), WOLF-PSORTtool (https://wolfpsort.hgc.jp/), ProtComp (http://linux1.softberry.com/berry.phtml), and CELLO v.2.5 (http://cello.life.nctu.edu.tw/) on 26 February 2022. Protein sequences were used as input files in the mentioned servers, which were retrieved from the online NCBI database (https://www.ncbi.nlm.nih.gov/) on 11 February 2022.

### 2.11. Protein-Protein Interaction Network Analysis

For further insight into the interaction of IMF-associated circRNA target genes, we ran a protein-protein interaction network on an online tool of a string database (http://string-db.org/) at 25 February 2022. As the input source, we provided the peptide sequences file of the top 300 differentially expressed target genes of our key circRNAs (circRNA_08840 and circRNA_23437) ranked by *p*-value < 0.05 and fold change ≥ 2. By using the STRING database (http://string-db.org/) on 25 February, the PPI network was constructed with the nodes and edges consisting of genes, and Cytoscape was used to visualize the network. The key genes were displayed prominently with a greater number of nodes and edges.

### 2.12. Real-Time Fluorescence Quantitative PCR Verification

Extracted RNA from IMF tissue of the longissimus dorsi muscle was acquired from two pig breeds (L_JN and D_JN) by using TRIzol (Invitrogen, Carlsbad, CA, United States), which was reverse-transcribed to produce cDNAs using Prime Script RT Reagent Kit (Perfect Real Time; TaKaRa, Osaka, Japan). According to the method of selecting DECs and DE miRNA in other relevant studies [42,43,53,54], we randomly selected 6 DECs and 3 DE miRNAs. Afterward, they were subjected to a quantitative real-time PCR (qRT-PCR) analysis on the Applied Biosystems 7500 Fast Real-Time PCR System (Roche, Basel, Switzerland; v 2.0.5) with SYBR Green qPCR SMix (ROX; Roche). The qRT-PCR analysis was used to validate the reliability of transcriptome data used in this study by comparing their expression profiles. Six CircRNAs (circRNA_23437, circRNA_14759, circRNA_23442, circRNA_09667, circRNA_08528, circRNA_08840); three miRNAs (ssc-miR-370, ssc-mi-339-3p, and ssc-miR-769-3p) were amplified with specific primers (Supplementary Table S1). MiRNA reverse primers are universal primers that were derived through the QIAGEN kit. The glyceraldehyde 3-phosphate dehydrogenase (GAPDH) gene, 5S, and beta-actin (ACTB) were internal references of circRNA, miRNA, mRNA, respectively. The 2-ΔΔCt method was used to determine the relative transcript abundance from different samples [55].

### 2.13. Protein Modeling for Structural Analysis

For further structural-functional analysis, full-length peptide sequences of key genes (obtained from the ceRNA co-expression network and verified from the PPI network and qRT-PCR analysis) were entered into the web interface of the Swiss model of the protein structure (https://swissmodel.expasy.org/) on 23 February to acquire a preliminary 3D structure of the required gene. The SWISS model web interface was used with default settings to acquire the model based on homology. The software returned with the highest scoring templates used to model the concerned protein sequences. The same was repeated through the web interface of the PHYRE2 Protein Fold Recognition Server [56] to confirm the reliability of our results. Models were carefully selected with criteria of the similarity index of at least 40% and 100% confidence levels. A representative model of each protein was downloaded in PDB format, which was further used in Chimera software for the annotation of specific regions/loops/chains or conserved sites 2.14. 

### 2.14. Statistical Analysis

All data presented here are mean values ± standard deviations. Student’s T-test was performed for the comparison, and *p*-value < 0.05 was considered statistically significant.

## 3. Results

### 3.1. Circular RNA Library Construction and Quality Control

**Figure 1:**
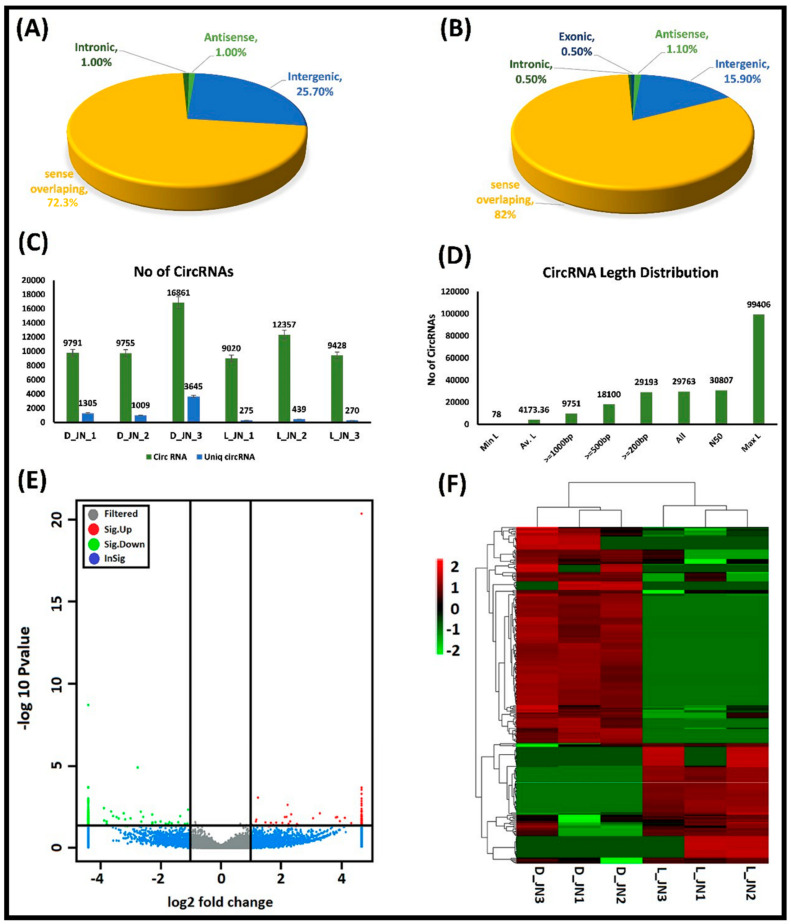
The proportions, characteristics, and distributions of various circRNAs identified in the IMF tissues of two pig breeds. Classifications of the DECs are given as: (**A**) upregulated and (**B**) downregulated; (**C**) represents the number of circRNAs predicted in L_JN and D_JN; (**D**) shows the length distributions of the identified circRNAs; (**E**) illustrates the upregulated circRNAs with red dots on the right side and downregulated with green dots, while grey represents the circRNAs with non-significant expression differences between both groups of pig breeds under |fold change| ≥ 2.0 and *p*-value < 0.05; (**F**) shows the cluster analysis of DECs. The expression data were clustered with a log10 (TPM + 1) value. The color scale indicates the expression of DECs: red and green indicate up- and downregulation, respectively. D_JN and L_JN represent two different pig breeds.

For comprehensive in-depth profiling of circRNAs in pigs, we performed a high-throughput transcriptome analysis of intra-muscular fat tissues from the longissimus dorsi muscles of two pig breeds—the Laiwu pig (L_JN) and Large White pig (D_JN). We went through strong quality control measures for our transcriptome data to ensure accuracy before starting the subsequent analysis. Initially, we identified a total of 480,082,001 raw reads (240626318 from D_JN and 239,455,683 from L_JN) from six RNA-Seq libraries. Removal of low-quality, poly-N, and adapters containing reads from the raw reads resulted in the identification of 463,803,620 clean reads, which consisted of 231,679,016 and 232,124,604 clean reads from the D_JN and L_JN samples, respectively (Figure 1C), and were mapped with reference genomes. A total of 29,763 circRNAs were supported by at least two junction reads identified from the longissimus dorsi muscle and spliced from 30,807.

### 3.2. Identification, Characterization, and Expression Profiling of circRNAs

The Laiwu pig (L_JN) and Large White pig (D_JN) have great differences in their meat quality, regarding IMF content, meat color, and tenderness (see the introduction for the details). Comparative tissue-specific expression profiling of both pigs will provide a good genetic source to identify potential circRNA and miRNA involved in the regulation of fat deposition and fat-related syndromes. To further explore the implications of circRNA and miRNA in IMF tissues, we examined the expression profiles of circRNAs in the longissimus dorsi muscles of the two mentioned pig breeds under |fold change| ≥ 2.0 and *p*-value < 0.05 as a selection criterion, which showed the significant differences among expression profiles of circRNAs (Figure 1E) and in the cluster heat map (Figure 1F). Furthermore, we identified 284 DECs (101 upregulated and 182 downregulated), of which, 35.7% of circRNAs had more than two-fold difference counts. From 101 upregulated DECs, 26 belonged to intergenic circRNAs, 1 from intronic, 73 were sense-overlapping, and 1 was antisense circRNA (Figure 1A). Among 182 downregulated circRNAs, there was 1 exonic circRNA, 29 intergenic, 1 intronic, 149 sense-overlapping, and 2 antisense circRNAs (Figure 1B). Furthermore, Figure 1C presents the overall picture of total and unique circRNAs retrieved from all of the samples of the two pig breeds. The lengths of the identified circRNAs ranged from a minimum of 78 bp to a maximum length of 99,406 bp (Figure 1D). The overall distributions of differentially expressed circRNAs in L_JN vs. D_JN intramuscular fat tissues are shown in Figure 1.

### 3.3. miRNA Library Construction and Quality Control

**Figure 2:**
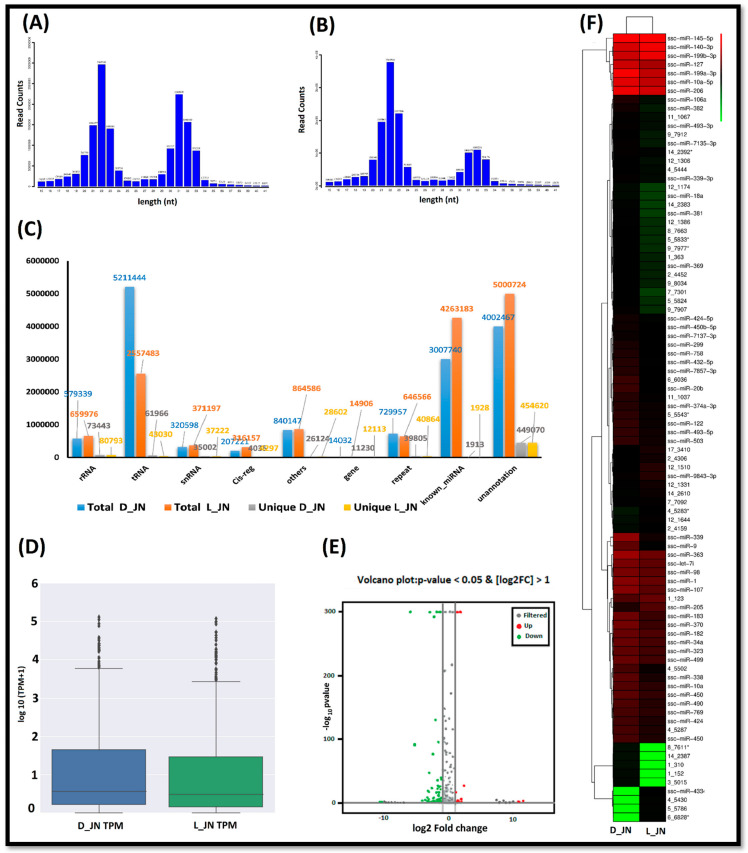
Statistics of miRNA in two contrasting pig samples; (**A**) length count of miRNAs in the D_JN pig; (**B**) length count of miRNAs in L_JN. (**C**) Comparison of various small non-coding RNAs in two pig breed samples. (**D**) Box plot generation analyzes the symmetry of miRNA data in L_JN and D_JN. (**E**) Volcano plot representing DEmiRNAs in the IMF tissue of L_JN and D_JN. The red dots and green dots represent the upregulated and downregulated miRNAs in IMF tissue, respectively. (**F**) Heat map of DEmiRNAs presenting their expression patterns in two pig samples between two color peaks (red represents upregulated and green portrays downregulated DEmiRNAs).

miRNA sequencing provided us with 16,460,254 and 15,785,824 raw reads, which were reduced to 14,912,945 and 14,694,778 clean reads after applying quality control measures from three of each of the D_JN and L_JN libraries, respectively. The length distributions of the clean reads illustrate the status of small RNA (sRNA) in the samples. Figure 2A, B demonstrate the typical length distributions for both samples from 21–25 bp peaks but at times they also show the peaks of 31–35 bp, which may be due to piRNA, tRNA, or rRNA. Moreover, clean reads were mapped to the reference genome and aligned reads were counted as 12,733,528 and 12,556,731 from D_JN and L_JN samples, respectively. Similarly, small RNAs were also recognized in a large number along with miRNA, tRNA (tiRNA, tRFs), rRNA, piRNA, snoRNA, etc. (Figure 2C). So far, 38,589 miRNA entries have been counted with the miRbase database (http://www.mirbase.org/) (22 November 2021), including 408 precursors and 457 mature pigs (Sscrofa10.2). In the current study, we predicted 370 novels and 271 known miRNAs in D_JN, with 367 novel and 276 known miRNAs in L_JN.

### 3.4. Identification, Characterization, and Expression Profiling of miRNAs

Similarly, a high differential screening standard with *p*-value < 0.05 and TPM difference multiple > 2 provided us with a total of 90 differentially expressed miRNAs (DEmiRNAs), including 19 upregulated and 71 downregulated (Figure 2D,E). The result revealed that 50 known miRNAs, including ssc-miR-127, ssc-miR-339, ssc-miR-339-3p, ssc-miR-370, ssc-miR-503, ssc-miR-7137-3p, and ssc-miR-769-3p were downregulated in the Laiwu pig as well as the Large White pig under the control group, while 40 novel DEmiRNAs were identified. The volcano map of DEmiRNAs is presented in Figure 2E. The heat map of differentially expressed miRNAs was generated to investigate their expression patterns during tissue-specific fat metabolism and regulation (Figure 2F).

### 3.5. Co-Expression Network Analysis of circRNA-miRNA Interaction

**Figure 3:**
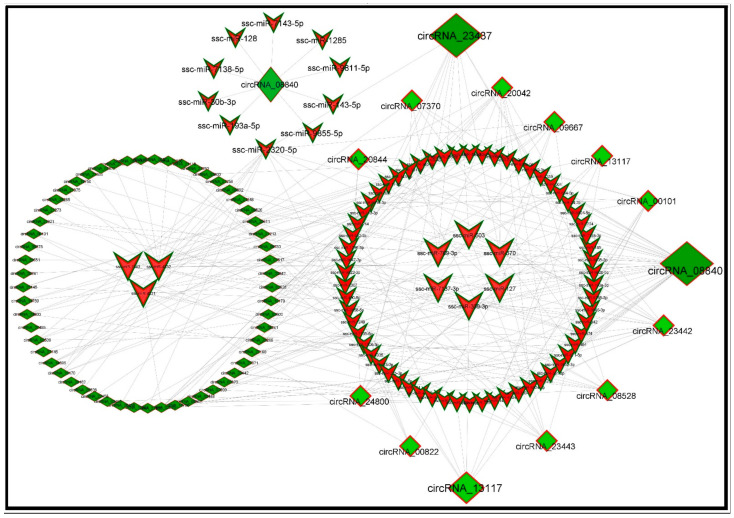
A network of differentially expressed circRNAs and their target miRNAs in intramuscular fat tissues. The square green nodes represent circRNA and the red triangular nodes represent miRNA. The increasing sizes illustrate the higher levels of interaction with each other.

circRNAs are considered very important for devouring regulatory roles in extensive-expression profiling, especially by limiting miRNA activity and RNA-binding proteins. miRNAs demonstrate their implications for normal growth, development, and homeostasis [26]. Thus, we retrieved the interaction of circRNA with their target miRNAs, which can be helpful to understand the functions and mechanisms of circRNA as a sponge of miRNA by establishing a regulatory network among them. We constructed a co-expression network of the top 300 circRNA-miRNA interacting pairs with smaller descending *p*-values. The MetScape package of Cytoscape software (v.3.8.2) was used to visualize the network of DECs with their target miRNAs from the IMF tissues of L_JN and D_JN pig samples. Our analysis showed that circRNA_23437 had the highest number of miRNA-binding sites, followed by circRNA_08840, circRNA_08528, circRNA_23442, circRNA_07370, circRNA_13117, and so on (Figure 3).

### 3.6. Interaction of ncRNAs with mRNA as ceRNA in IMF Tissues

**Figure 4:**
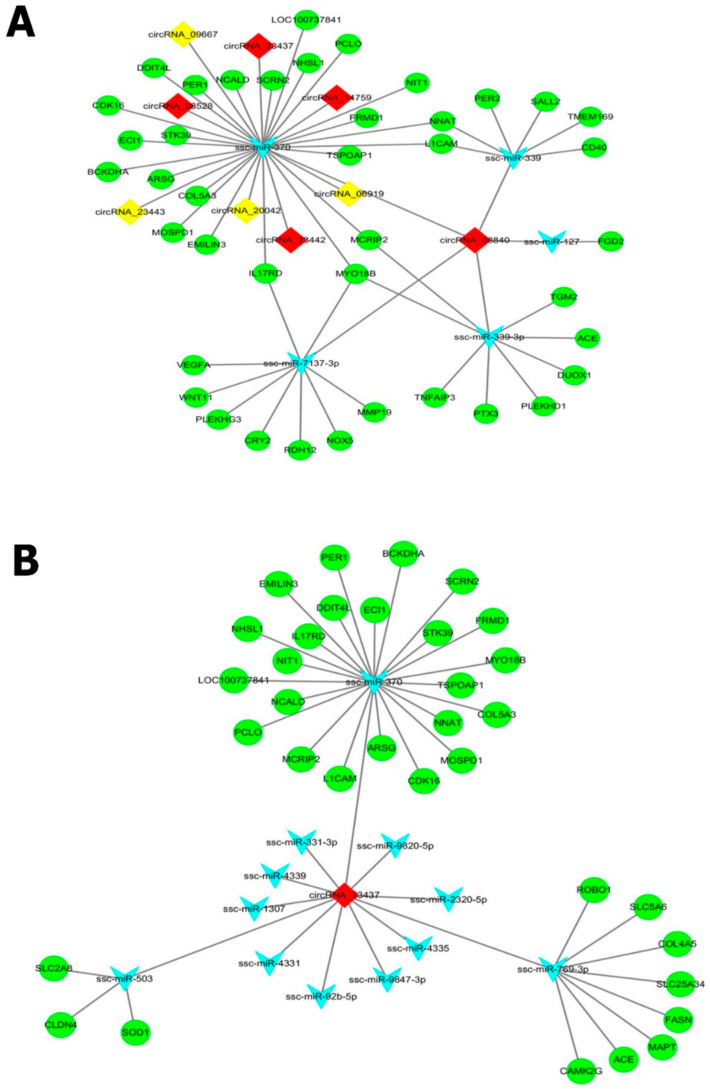
After the identification of two lipid metabolism-associated circRNAs, a ceRNA network was constructed from these two circRNAs through a regulatory network of circRNAs, miRNAs, and mRNAs. (**A**) circRNA_08840 binds to 5 miRNAs and their target genes. (**B**) circRNA_23437 binds to 12 miRNAs and their target genes. Note: the red color represents upregulated circRNAs, yellow represents downregulated circRNAs, blue denotes miRNAs, and the green represents mRNA.

According to the competitive endogenous RNA (ceRNA) hypothesis, RNA transcripts, such as circRNAs, mRNAs, and miRNA response elements (MREs) are in competition with each other for miRNA-binding (to regulate the expressions of each other. Following the “ceRNA hypothesis”, we constructed a ceRNA regulatory network (Figure 4) by integrating the expression profiles and regulatory relationships of the circRNAs, miRNAs, and mRNAs. The network was visualized in the MetScape package of Cytoscape (v.3.2.1) software for visual observations. To be more specific and for precise results, we only selected two upregulated key circRNAs containing the largest modules with the maximum number of target miRNAs. Of them, circRNA_23437 harbored 12 and circRNA_08840 encompassed 5 miRNA-binding sites interacting with their differentially expressed genes. As expected, all target miRNAs showed downregulated expression patterns (Table 1).

Five miRNAs (sscmiR-7137-3p, sscmiR-370, sscmiR-339, sscmiR-127, and sscmiR-339-3p) were found on the binding sites of CircRNA_08840 followed by the identification of three miRNAs (Ssc-miR-370, ssc-miR-503, and ssc-miR-769-3p) on the binding sites of circRNA_23437. These miRNAs were differentially expressed in the intramuscular adipose tissues of the Large White and Laiwu pigs (Table 2). The remaining miRNAs on the binding sites of the mentioned circRNAs (as shown in Figure 4A,B) were not differentially expressed, but they may be the potential targets of the mentioned circRNAs. It is quite surprising in our results that miRNA “Ssc-miR-370” is associated with both of our key circRNAs as a common target binding site of CircRNA_08840 as well as circRNA_23437. In L_JN IMF tissue, expressions of differentially expressed circRNAs were upregulated; corresponding miRNA expressions were downregulated (Table 2).

### 3.7. Functional Enrichment Analysis of DEC Host Genes

**Figure 5:**
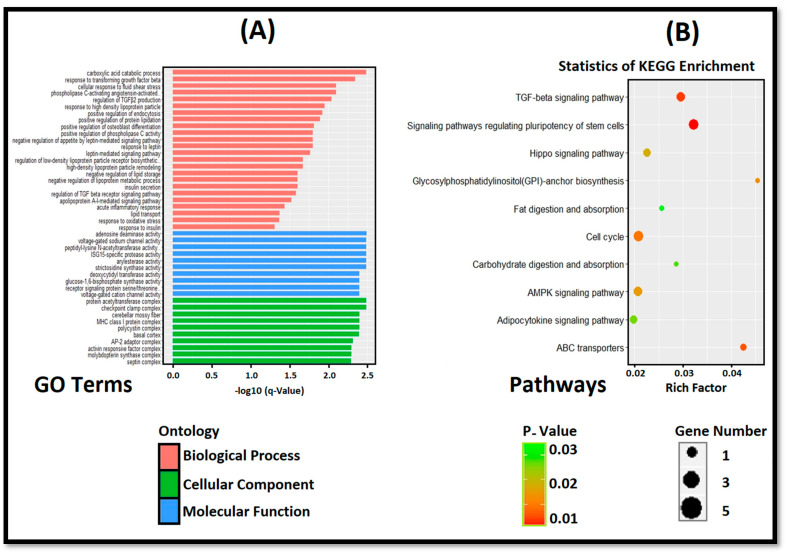
Functional enrichment analysis of host genes (**A**) shows the GO analysis for host genes of DECs; (**B**) demonstrates the KEGG pathway enrichment analysis for host genes of DECs. Note: the names of the pathways are shown on the *Y*-axis and the enrichment factor on the *X*-axis. The sizes and colors of the circles represent the quantity and significance of DEC-enrichment in a certain pathway.

In the present study, a total of 631 differentially expressed circRNA host genes were identified in the intramuscular adipose tissues of the L_JN and D_JN; the GO enrichment analysis revealed that these genes were involved in lipid metabolism. According to the biological process, they were mainly involved in carboxylic acid catabolism, the response to leptin, the cellular response to fluid shear stress, transcriptional growth factor response, high-density lipoprotein particle reaction, insulin resistance, lipid transport, negative regulation of lipid storage/appetite by leptin-mediated signaling pathways, positive regulation of endocytosis, regulation of TGFβ2 production, regulation of the TGFβ signaling pathways, and acute inflammatory response (Figure 5A). Under the molecular functions category, DEC host genes were found highly enriched in adenosine deaminase activity, the voltage-gated sodium channel activity, peptidyl-lysine N-acetyltransferase activity, ISGI-specific protease activity, receptor signaling protein serine/threonine, and glucose-1,6-biphosphate synthase activity. In the cellular component, the mentioned DECs were found abundantly in the acetyltransferase complex, cerebellar mossy fiber, MHC class I protein, AP-2 adaptor complex, polycystin complex, septin complex, basal cortex, and the molybdoprotein synthase complex (Figure 5A).

The KEGG enrichment analysis showed that DEC host genes were enriched into 144 signal pathways, among which, 10 were screened with a *p*-value < 0.05, as shown in Figure 5B. Differentially expressed circRNA host genes were significantly enriched in pathways of potential stem cell signaling, transcriptional growth factor, adipocytokine, cell cycle, fat metabolism, and other signal pathways related to adipogenic differentiation and lipid metabolism. The KEGG enrichment results are consistent with the results of the GO enrichment analysis (Figure 5B).

### 3.8. Functional Enrichment Analysis of DEC-Target Genes

**Figure 6:**
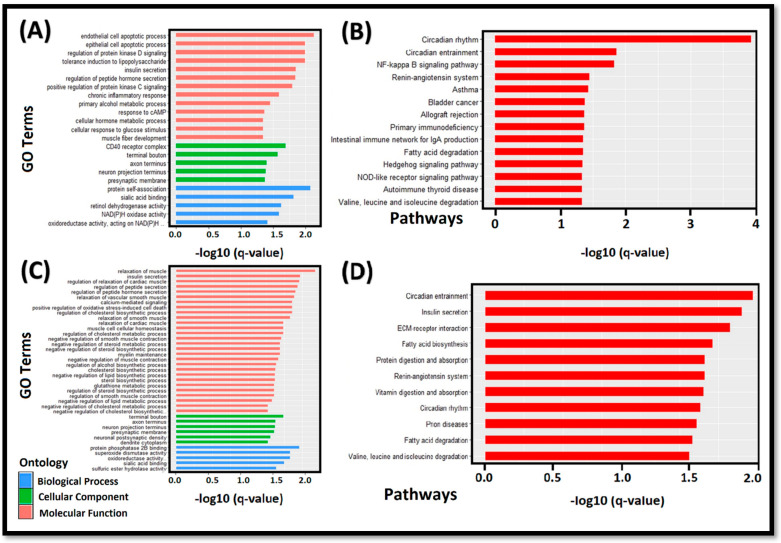
Functional enrichment analysis of the target genes; (**A**,**B**) represent the GO and KEGG pathway enrichment analyses of the target genes of circRNA_08840, respectively; (**C**,**D**) display the GO and KEGG enrichment results for circRNA_23437-associated target genes, respectively.

circRNAs can regulate the functions and expressions of their target genes, acting as miRNA sponges by modulating their transcription, regulating protein activity, mRNA stability, and degradation [57]. Furthermore, circRNAs can regulate the expressions of target genes by various regulatory mechanisms or indirect pathways involved in fat metabolism. For an in-depth functional characterization of our circRNA-based target genes, we performed a comprehensive gene ontology and KEGG pathway enrichment analysis to further explore the underlying molecular mechanisms of the lipid deposition. Circular RNA (circRNA_08840)-associated target genes composed of GO terms in the biological process (BP), cellular component, and molecular function are shown in Figure 6A. The significant GO terms for circRNA_08840 target genes were as follows: endothelial and epithelial cell apoptosis in BM, CD40 receptor complex in the cellular component, and protein self-association in the molecular function. The KEGG pathway analysis for target genes of circRNA_08840 was significantly enriched in the nuclear factor kappa-light-chain-enhancer of activated B cells (NF-kappa B signal pathway), the hedgehog signal pathway, and NOD-like receptor signaling pathways (Figure 6B). Similarly, important enriched GO terms for circRNA_23437 target genes were as follows: muscle relaxation in BM, terminal button in a cellular component, and protein phosphatase 2B-binding in the molecular function (Figure 6C). The KEGG pathway analysis for target genes of circRNA_23437 was significantly enriched in the circadian rhythm, insulin secretion, ECM receptor interaction, fatty acid biosynthesis, protein digestion and absorption, renin-angiotensin system, and fatty acid degradation. Moreover, abdominal fat contains appreciable amounts of infectious prions (Figure 6D). Differentially expressed miRNAs may regulate the pathways of differentially expressed target genes and ultimately alter their signal transductions. In animals, miRNAs are not fully complementary to their target mRNA, mainly targeting the 3’ non-coding region (3’ UTR) of the target mRNA and acting as mechanisms to inhibit the translation [43,54]. circRNAs, such as ceRNA, work as miRNA sponges and control the functions of miRNAs, indirectly regulating the expressions of the target genes.

### 3.9. Expression Profiling of Target DEGs, Gene Structure, and Subcellular Localization

**Figure 7:**
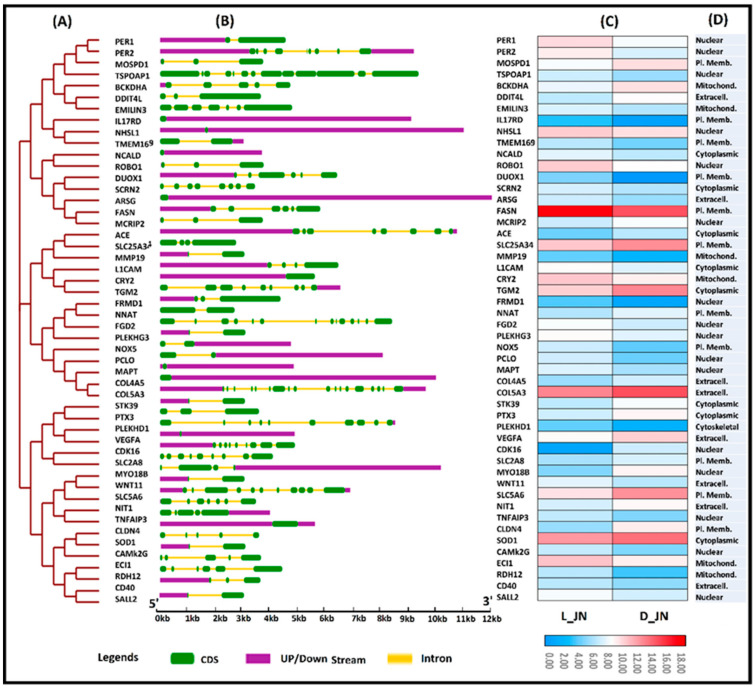
Relationship between gene structures, subcellular localization, and expression patterns among differentially expressed target genes of IMF-associated CircRNAs; (**A**) represents the phylogenetic tree built with the neighbor-joining method and 1000 bootstrap replicates; (**B**) shows the gene structure of target genes containing different numbers of exon and introns; (**C**) demonstrates the expression profiles of IMF-associated target genes based on FPKM values; (**D**) shows the subcellular localization of all target genes.

In animals, most genes are interrupted by one or several exons and introns. Their arrangements can be used to analyze their evolutionary associations among each other and provide some clues about their involvement in a particular function [58]. To gain further insight into the evolutionary, structural, and functional association of target genes, we organized a comprehensive comparative study of these genes by constructing a phylogenetic tree and portrayed these genes by comparing gene structures, tissue-specific expression patterns, and their predicted subcellular localizations in the same layout. Figure 7A–D represent the powerful illustration of this schematic study on how gene structures and subcellular localizations are interlinked with each other along the phylogenetic tree, with common evolutionary trajectories following similar functions represented by analog expression profiles. 

**Figure 8:**
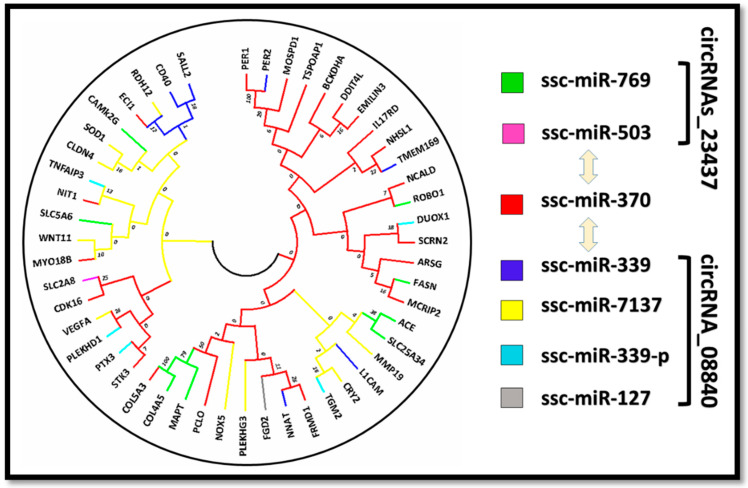
Two rooted phylogenetic trees constructed with the neighbor-joining method. Bootstrap values from 1000 replicates are indicated at each node of the tree. The tree shows various clades representing separate phylogenetic groups in each clade but similar genes in the same clade. Different colors in the tree represent the target genes of different miRNA. Ssc-miRNA-370 was found in both circRNAs as a common binding site.

Associations of exon-intron distribution patterns with their biological functions have been witnessed in previous studies [59]. In the current study, ARSG was the longest gene with a length of 12 kb compared to (NNAT), which had the shortest genomic length of 3 kb. For further insight into the structural evolution of these genes, we developed a new phylogenetic tree and examined their structural features using the online web portal GSDS (http://gsds.cbi.pku.edu.cn/) on 27 February 2022. A comprehensive analysis of exon-intron structures aligned into the phylogenetic tree sequence shows their relative lengths (Figure 7B) and demonstrates their random distributions throughout the tree; however, their clustering into similar clades was observed for genes with similar structures. The best representation was exhibited by ss-mir-370, which was common in both circRNAs (cirRNA-23437 and cirRNA-08840) (Figure 7A and Figure 8). None of the exon regions varied among the genes (from 1 to 18); however, surprisingly, all closely associated genes exhibited similar structural organizations and diverged in exon-intron lengths. To elucidate the potential differences in IMF regulation and deposition between two groups of interest (L_JN and D_JN), we compared the mRNA expression profiles of the target genes involved in ceRNA regulatory network (Figure 7C). Protruding differences were witnessed in the gene expression profiles between D_JN vs. L_JN. FASN and COL5A3, with the target genes of ssc-miR-769-3p being highly expressed and upregulated in the intramuscular adipose tissue of Laiwu pigs compared to Large White pigs. Similarly, differential expressions of ECI1, SLC24A34, SLC5A6, SOD1, TGM2, FASN, PER1, and COL5A3 were observed during the tissue-specific expression analysis of IMF tissues from both groups. It is evident that our results from the tissue-specific expression profiling are in agreement with results from the ceRNA network, GO analysis, and KEGG enrichment analysis, which obviously ‘approved’ the reliability and accuracy of our transcriptome data analysis, and was further confirmed by the pRT-PCR analysis.

For the subcellular localization prediction of IMF-associated target genes, we used several websites and online tools to ensure the accuracy of results, as presented in the methodology section. Taking all the results together, we found that most of the mentioned genes were found in the plasma membrane, mitochondria, and cytoplasm. Few of the genes were also predicted in nuclear and extracellular membranes. This is consistent with their associated functions (Figure 7D). It has been observed that most of the CircRNA_23437-based target genes were grouped in the same clades and contained similar subcellular localization along with similar expression profiles; similar results were also observed for CircRNAs_0884. However, ssc-mir-370 was identified as a common miRNA-binding site of both key circRNAs (circ_23437 and CircRNA_0884) and contained similar target genes for both circRNAs, signifying the accuracy and reliability of our transcriptome data analysis, portraying a strong association of gene structures and their subcellular localizations with tissue-specific expression patterns along the phylogenetic tree (Figure 7 and Figure 8).

### 3.10. Protein-Protein Interaction Network Analysis for DEGs

Protein-protein interaction networks (PPIN) are the physical connections established between multiple numbers of proteins with greater specificity as a result of biochemical processes directed by different interactions, including electrostatic forces, hydrogen bonding, and the hydrophobic effect. As their functions tend to be regulated, proteins rarely act solely. Many molecular processes within a cell are carried out by molecular machines that are built from numerous protein components organized by their PPIs. Following the PPI network hypothesis, we also constructed a PPI network among the proteins of the top 300 DEGs acquired from our comparative transcriptome analysis of IMF tissues of two pig breeds. We used protein sequences of the mentioned DEGs as an input file in the string database to obtain in-depth insights into the interacting proteins to regulate similar functions on a common floor. Thus, we could approach the potential key genes involved in the regulation of common genes.

**Figure 9:**
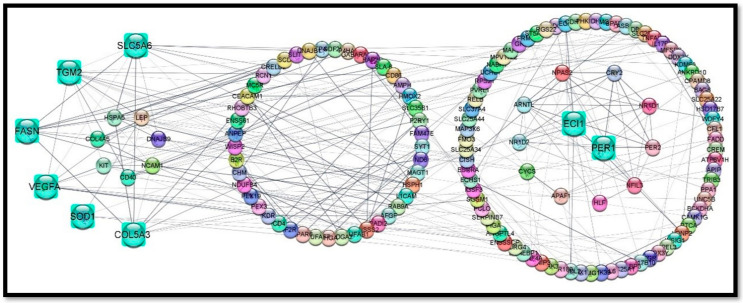
Protein-protein interaction network between the top 300 downregulated and upregulated DEGs in the IMF tissues of L_JN and D_JN by threshold *p*-value < 0.05 and fold change ≥ 2.

This approach seems to be working amazingly, as we found that various key potential genes acquired from the ceRNA regulatory network analysis of transcriptome data were also found as hub genes in the PPI network. Importantly, ECI1, TGM2, SLC25A34, PER1, COL5A3, SLC5A6, SOD1, and FASN exhibited strong interactions with other genes (Figure 9). The expressions of these genes were regulated by miR370, miR769-3p, and miR339-3p. Of note, a variety of studies exposed the role of the mentioned hub genes in fat metabolism and regulation.

### 3.11. Structural-Functional Relationship of IMF-Associated Key Genes

The organization of atoms into a three-dimensional structure is responsible for the unique biological functions associated with any protein. It may be due to the specific organization of catalytic residues in an active site, the various interactions within the same protein, or among different proteins for numerous regulatory and structural purposes. The availability of the protein’s three-dimensional structure is a great source for an in-depth understanding of the functional mechanisms and dimensions that facilitate the development of a hypothesis about its possible role. It further illustrates the consequences of any required modification in the structure and how to regulate its functions. For example, knowing a protein’s structure could allow us to design site-directed mutations with the intent of changing the functions.

**Figure 10:**
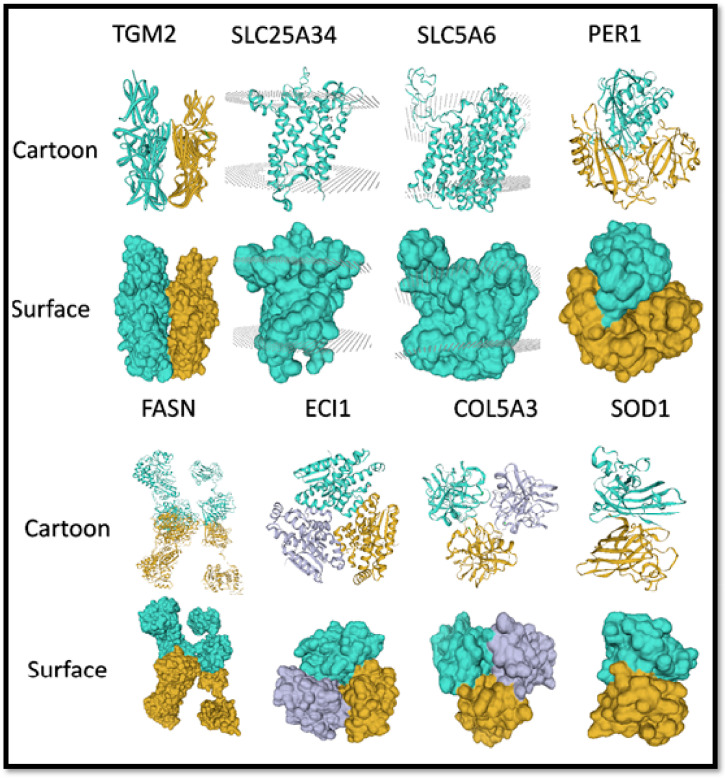
A 3D model structures of eight IMF-associated proteins in cartoon and surface orientations. Models were selected on homology bases with at least 40% similarity and a 100% confidence level.

The functions of any protein are associated with its unique structure. So, for all protein design studies, the most important thing is to determine its precise structure. X-Ray crystallography has been a more commonly used technique; obtaining protein structure information is a routine, highly automated procedure. Due to the higher level of complexity and long-time span to acquire the experimental structures of the required proteins through protein NMR and X-ray crystallography, homology protein modeling is the best source to generate hypotheses for function-specific proteins and direct further experimental work. Taking together all of the above reasons and the significance of protein modeling, we constructed 3D protein models of the above-discussed key genes that we screened from a ceRNA regulatory network and PPI network for an in-depth insight into their structural-functional relationship, based on protein homology modeling, as shown in Figure 10.

We entered the full-length peptide sequences of key genes into the web interface of the Swiss model of the protein structure (https://swissmodel.expasy.org/) 27 February 2022 to acquire preliminary 3D structures of the required gene. The SWISS model web interface was used (at default settings) to acquire the model based on homology. The software returned with the highest scoring templates used to model the concerned protein sequences. The same was repeated through the web interface of the PHYRE2 Protein Fold Recognition Server [56] to confirm the reliability of our results. Models were carefully selected with criteria of the similarity index, with at least 40% and 100% confidence levels. A representative model of each protein was downloaded in PDB format and was further used in Chimera software for the annotation of specific regions/loops/chains or conserved sites (Figure 10).

### 3.12. qRT-PCR Verification

**Figure 11:**
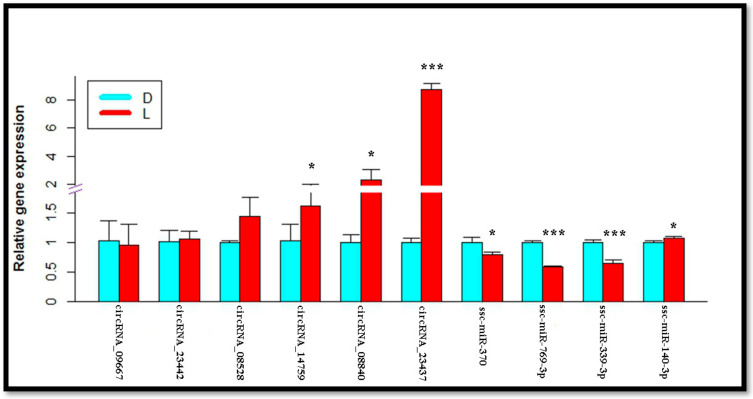
The qRT-PCR verification result of differentially expressed circRNAs, miRNAs in the longissimus intramuscular adipose tissues of the Large White and Laiwu pigs (L_JN vs. D_JN). Significance of the correlation: * means *p* < 0.05, *** means *p* < 0.001.

Based on previously determined results of miRNAs and mRNAs from IMF tissues of Large White and Laiwu pigs [26,60,61], as well as their circRNA-associated target miRNAs, we went through a qRT-PCR analysis of six DECs to verify our predicted results. this showed that ssc-miR-370, ssc-miR-769-3p, and ssc-miR-339-3p exhibited significant downregulation in the intramuscular adipose tissue of the Laiwu pig. Further, circRNA_09667 was not significantly upregulated in the intramuscular adipose tissues of the Large White pigs. However, circRNA_14759, circRNA_23437, and circRNA_08840 were significantly upregulated in the L_JN intramuscular adipose tissue; circRNA_23442 and circRNA_08528 were not significantly upregulated in the L_JN intramuscular adipose tissue (Figure 11). The above findings are consistent with our predicted results from the transcriptome data. Although circRNA_25555, circRNA_09667, and circRNA_23442 have no significant differential expressions, their expression trends are consistent, indicating the reliability of our transcriptome data for mining genetic material associated with IMF tissues.

## 4. Discussion

### 4.1. Significance of Intramuscular Fat Deposition in Pork 

Fats and lipids contribute a great deal when defining meat quality because of their associations with sensory attributes and nutritional value for human consumption [5]. IMF in pork is associated with better meat quality in terms of tenderness, juiciness, and visual characteristics [62,63]. However, abnormal fat depositions are also linked with a wide range of diseases that may produce different disorders in humans [64,65]. Genetic selection programs have magnificently reformed the body compositions of livestock but further in-depth insight is required into the molecular regulation of IMF and the development of adipocytes to enhance production efficiency without any adverse impacts on the sensory aspects of muscle food [66]. A series of transcriptomic studies were conducted to identify genes associated with pathways involved in fat regulation [62,67]. Implications of circular RNA as competitive endogenous RNA in IMF tissue remained unknown for a very long time. The pig is recognized as a biomedical model of type 2 diabetes, obesity, and associated metabolic syndromes in humans [17]. Therefore, exploring the key circular RNA acting as a microRNA sponge, the target genes and pathways associated with IMF depositions in pigs may provide valuable information that is helpful from a medical aspect.

For the first time, this study examined the potent role of circRNA as a competitive endogenous RNA (ceRNA) in the IMF tissue of the longissimus dorsi muscle between Large white pigs (D_JN) and Laiwu pigs (L_JN). The purpose was to classify the potential role of circRNA related to lipid metabolism and understand the biological and molecular mechanisms underlying fat deposition. The interactions of mRNA, miRNA, and circRNA were also noticed in the adipose tissues of different pig breeds and the upregulation of ssc_circ_0002807 and ssc_circ_0005382 in the intramuscular tissue, and may be involved in fat deposition and lipid metabolism. Meanwhile, ssc_circRNA_11897 and ssc_circ_26852 were observed upregulated in the subcutaneous adipose tissue of the pig breed and involved in the pathways related to adipocyte differentiation and lipid metabolism [26]. Similarly, in the current study, we documented 283 differentially expressed circRNAs from IMF tissues of the longissimus dorsi muscle of two pig breeds, of which 101 circRNAs were upregulated and 182 circRNAs were downregulated (Figure 1).

Functional enrichment analyses of differentially expressed circRNA host genes were significantly enriched in fluid shear stress, fat metabolism, high-density lipoprotein particles, protein lipids, leptin reaction, lipid storage, insulin secretion, and so on (Figure 5A). Leptin is the first hormone produced by adipocytes [68], which regulates appetite and achieves energy balance by acting on receptors in the arcuate nucleus of the hypothalamus [69]. The KEGG pathway analysis was significantly enriched for the carboxylic acid catabolic process, the TGF-beta signaling pathway, AMPK signal pathway, fat factor signal pathway, and so on (Figure 5B). AMPK is a key sensor of the cellular energy state, which may participate in glycogen metabolism and lipid metabolism. It is reported that the activation of the AMPK signaling pathway can inhibit the differentiation of 3T3L1 preadipocytes and adipogenesis [70]. Polyphenols extracted from Rosa plants can regulate lipid metabolism by activating the AMP-activated protein kinase (AMPK) signal pathway [71]. It has also been reported that the Hippo signal pathway [72], TGF-beta signal pathway [39], and the fat factor signal pathway relate to lipid metabolism [40]. In fact, TGF-β is also famous for the regulation of fibrosis and recently it was also revealed that TGF-β increases collagen type I expression in both myoblasts and differentiated myotubes via extracellular matrix preservation, enhancing the matrix protein [73]. As a result, the expression of ECM enhanced collagen deposition, which is mainly important for the network buildup of intramuscular tissue [74,75]. Moreover, Zhang et al. reported that miR-140-3p can regulate adipocyte differentiation by targeting TGF-β1 [35]. In addition, TGF-β1 can phosphorylate SMAD3. The activated SMAD3 can bind C/EBPs to inhibit their transcriptional activity and, thus, it plays a role in the early stage of adipocyte differentiation [36].

### 4.2. Crosstalk of circRNAs with Their Target miRNAs Stimulating Fat Metabolism

It should be known that circRNAs are endogenous transcripts with multiple MREs (miRNA response elements) that can interact with the miRNA commonly known as “miRNA sponges” [48,76], which attenuate the targeted inhibition of downstream mRNAs by miRNA as ceRNA and inevitably regulate the expressions of protein-coding genes [67,77]. An in vitro study on mice revealed the contribution of CircRNA_010567 in myocardial fibrosis via sponging miR-141, which regulates the expression of transforming growth factor-beta 1 (TGF-β1) [38]. In our study, to explore the role of circular RNAs in lipid metabolism as ceRNAs, their target MREs were predicted by constructing a co-expression network between DECs and their target miRNAs (Figure 3). In the co-expression network analysis of circRNA-miRNA, circ_08840 and circ_23437 were identified as key circular RNA along with their differentially expressed target miRNAs.

In circRNA_08840, 48 potential binding sites for target miRNAs were witnessed; however, ssc-miR-370, ssc-miR-127, ssc-miR-339, ssc-miR-339-3p, and ssc-miR-7137-3p showed higher levels of differential expression among them (Figure 3, Table 1). Similar results were reported by Gao et al. (2012), who found higher expression levels of miR-370 in patients with hyperlipidemia than in normal subjects, and the expression of miR-370 was positively correlated with the expressions of cholesterol, triglyceride, low-density lipoprotein, and high-density lipoprotein in blood, and positively correlated with the severity of coronary artery disease [78]. Similarly, Iliopoulos et al. (2010) found that miR-370 can upregulate the expressions of liver lipid metabolism-related genes SREBP-1c, DGAT2, and ACC1, and participate in the biosynthesis of fatty acids and triglycerides, which impact meat tenderness [46]. Li et al. reported that miR-370 can oxidize the low-density lipoprotein receptor OLR1, inhibit the translation of the OLR1 gene, and affect the formation of lipid droplets in the adipose cytoplasm [79]. miR-127 can promote myogenic cell differentiation by acting on S1RP3. Overexpression of miR-127 in amyotrophic mice can improve the disease phenotype, inhibit breast cancer cell proliferation, and promote apoptosis in humans [80].

The above-mentioned miRNA sponges, being the binding sites of circRNA_08840, have been involved in fat regulation and deposition, such as lipid metabolism, apoptosis, cell proliferation, inflammation, and differentiation. We propose that all of these functions are mainly controlled by the novel key circRNA_08840 reported in our study. Additionally, circRNA_23437 has potential binding sites for 12 miRNAs. However, sc-miR-370, ssc-miR-503, and ssc-miR-769-3p have earned the title of differentially expressed miRNAs. Of note, studies have explained that the overexpression of miR-503 can locally alleviate the inhibitory effect of TNF-α on myotube formation [81]. TNF-α is an inflammatory cell chemokine that generally has a high expression level in chronic diseases or muscle disorders; high levels of TNF-α can cause muscle atrophy. miR-503 (targeting CCND1) can be used as a tumor suppressor for esophageal squamous cell carcinoma. Downregulating miR-503 can promote invasion, cell proliferation, and migration. Migration and abnormal proliferation of vascular smooth muscle cells are very common in atherosclerotic disease progression. miR-503 inhibits the proliferation and migration of vascular smooth muscle cells induced by platelet-derived growth factors by acting on the insulin receptor (INSR) [82]. miR-503 is also reported to be involved in the regulation of cardiovascular diseases, such as myocardial fibrosis [83]. miR-769-3p is involved in the regulation of NDRG1 functions; overexpressed miR-769-3p has shown significant inhibition of cell proliferation, promoting apoptosis [84]. It has also been reported that miR-769-3p is associated with atherosclerotic diseases [85]. It can be assumed that circRNA_23437 and circ_08840 as ceRNAs might be key risk factors involved in lipid pathogenesis, with potential functions in lipid metabolism. These key circular RNAs are differentially expressed, affecting and modifying the expressions of target miRNAs, which are involved in a variety of pathways that affect fat regulation and deposition. In this study, the identified key circular RNA (08840 and 23437) expressions were upregulated and their corresponding target miRNA expressions were downregulated. Interestingly miRNA-370 was found to be a common target of both key circRNAs (Table 1 and Table 2).

### 4.3. Coherence of the ceRNA Regulatory Network with Differential Expression Datasets to Regulate Fat Metabolism

CircRNAs, as competing endogenous RNAs (ceRNAs) with miRNA response elements (MREs) and mRNAs, can competitively bind the same miRNA [26]; thereby, they regulate each other. This interaction of circRNA-miRNA and mRNA, commonly known as ceRNA, was analyzed by a co-expression network analysis to gain further insights into the regulatory mechanisms for potential genes associated with fat metabolism and regulation (Figure 4). The biological functions of ceRNA network-associated circRNA-targeted genes were also explored through the functional enrichment analysis (Figure 6). GO terms for circRNA_08840-associated target genes were enriched in apoptosis, the phosphokinase D signal, insulin secretion, chronic inflammation, muscle fiber development, and other biological processes (Figure 6A). GO terms of circRNA_23437-associated target genes were enriched in the following biological processes, e.g., muscle relaxation, regulation of cholesterol biosynthesis and metabolism, insulin secretion, smooth muscle contraction, and hormone secretion (Figure 6C). KEGG enrichment analysis of circRNA_08840 confirmed its involvement in NF-KB, Hedgehog, fatty acid degradation, and signaling pathways (Figure 6-B). circRNA_23437 was found involved in the circadian rhythm, insulin secretion, and fatty acid biosynthesis (Figure 6D). Of note, studies described the involvement of these pathways in fat regulation and metabolism. Any dysregulations in their normal functions lead to serious metabolic disorders [44,86,87,88].

Tissue-specific expression profiling of all corresponding target genes from the ceRNA regulatory network also supports these findings, and we can presume that the identified circular RNAs in the ceRNA network may play a central role in lipid metabolism, which is coherent with results from the protein-protein interaction network. Similar findings were concluded by [89] who observed that IMF and cholesterol deposition-related mechanisms are associated with the upregulation of genes involved in lipid and cholesterol syntheses. Eight key hub genes were identified from two independent studies, one based on the correlation network analysis of cirRNA-associated target genes using their expression data (Figure 4) and the second from protein-protein interaction networking using protein sequences from the top 300 DEGs (Figure 9). They include ECI1, SLC25A34, TGM2, SLC5A6, COL5A3, FASN, PER1, and SOD1. CircRNA functions as a microRNA (miRNA) sponge in the regulation of mRNA expression, forming the circRNA-miRNA regulatory axis. An integrated analysis of this study revealed the following circRNA-miRNA-mRNA regulatory axis, including the circRNA_23437-ssc-miR-769-3p-FASN axis, circRNA_23437-ssc-miR-769-3p-SLC5A6 axis, circRNA_08840-ssc-miR-339-3p-TGM2 axis, circRNA_08840-ssc-miR-503-SOD1 axis, and circRNA_08840-ssc-miR-769-3p-SLC25A34 axis (Figure 4)). Interestingly, both of our key circRNAs contained few binding sites for common miRNAs, such as circRNA_08840_23437-ssc-miR-370-ECI axis, circRNA_08840_23437-ssc-miR-370-COL5A34 axis, and circRNA_08840_23437-ssc-miR-370-PER1 axis. It can be hypothesized that through the above-mentioned circRNA-miRNA-mRNA regulatory axis, circRNAs as ceRNAs might be involved in IMF regulation, deposition, and meat quality, and associated with lipid metabolic syndrome in the case of ectopic IMF.

### 4.4. Significance of IMF-Associated Key/Potential Genes in Regulating Various Lipid Metabolic Syndromes

Enoyl-coenzyme isomerase (ECI1) is identified as a target gene of ssc-miRNA-370 in the current study, but has been known as a necessary coenzyme in the oxidation of unsaturated fatty acids and plays an important role in the oxidation of unsaturated fatty acids in mice [90]. Unsaturated fats contribute a lot to meat quality and consumer health [8,9]. Generally, the oxidation of lipids has a negative effect on meat due to the loss of essential fatty acids and vitamins, which reduce the nutritional value and sensory attributes [10]. Several studies have suggested that lipid oxidation contributes to the development of a pleasant aroma [2,6,11]. The literature shows that phospholipids are essential in the development of lipid oxidation, and lean meat is more susceptible to oxidation than fatty meat due to low marbling [12]. In the Lu et al. (2017) study on pigs, the authors determined that higher IMF content in fat-type pigs might be due to an increased need for ECI1-associated lipogenesis in the longissimus dorsi muscles and eventually affect meat quality [13,14]. Studies emphasized that the rise in lipogenesis, fatty acid uptake, fatty acid esterification, and the decline in lipolysis and fatty acid oxidation, contribute to enhanced IMF deposition in cattle and improve beef quality [15]. Lipids, such as unsaturated ester acyl and dicarboxylic acid urine, were observed accumulated in the livers of ECI1 knockout mice [91]. In this study, we found that ECI1 was upregulated in the IMF tissues of the L_JN pig, while the expression of ssc-miRNA370 was downregulated, which interacted with circRNA-08840 and CircRNA-23437, as shown in Figure 4. Furthermore, the ceRNA regulatory network analysis and expression profiling of DECs demonstrate that circRNA_23437 and circRNA_08840 sponge the activity of miRNA-370 and control the regulation of ECI1 gene expression through the circRNA-miRNA-mRNA regulatory axis, implying that both the key circRNAs might be involved in lipid metabolism by targeting ECI1 via sponging miRNA-370.

The SLC5A6 gene belongs to the solute carrier family and is regulated by ssc-miR-769-3p, which plays a vital role in the regulation and absorption of the water-soluble vitamin biotin (B7) and pantothenic acid (B5) in eukaryotes [92]. Both B7 and B5 are involved in the production of fatty acids, hormones, and cholesterol [93]. The deficiency of B7 may adversely affect lipid metabolism by inducing inflammation and immunologic disorders in mammals [94,95,96]. B5 is a pioneer of coenzyme A involved in fatty acid metabolism [97]; it plays an important role in the regulation of cellular oxidative stress [19]. Its deficiency may cause hypoglycemia, which often relates to the treatment of diabetes [97]. Similar results were witnessed by Thanh et al. (2022), who determined that higher SLC5A6 mRNA abundance might allow the efficient uptakes of vitamins and nutrients by the skeletal muscle and is responsible for membrane stability. Previously, researchers reported a positive impact of vitamin E on pork quality [20,21], beef quality [22,23], and the reduction of drip loss from pork chops by inhibiting the phospholipase A_2_ activity and lipid oxidation. Stimulation by Ca^2+^ phospholipase A_2_, undergoing hydrolysis, resulted in the formation of long chain unsaturated fatty acids. Increased phospholipase A_2_ activity noticed in pigs is susceptible to malignant hyperthermia (MH), responsible for producing excess Ca^2+^, leading to the formation of pale, soft, exudative meat (PSE meat) [23]. In this study, the expression of the SLC5A6 gene was observed upregulated in the L_JN pig targeted by miR-769-3p, exhibiting a downregulated expression. circRNA 23,437 (being a sponge for miRNA-769-3p) was implicated in the signal pathway related to vitamin digestion and absorption, which may associate with membrane stability and lipid metabolism. However, the exact role of SLC5A6 in IMF needs further investigation for validation.

Transglutaminase 2 (TGM2), as a target gene of ssc-miR-339-3p, is a multifunctional protein and has implications in various events, spanning wound healing and cell differentiation, to signal transduction and apoptosis [98]. In animals, TGM2 contributes to intramuscular adipogenesis and IMF content [24] and is widely applied in the meat industry (to improve the nutritional value of pork meat products) [25]. Adipogenesis has been negatively regulated in TGM2, lacking embryonic mouse fibroblast (TGM2-EMB) [99], which is in agreement with our findings on TGM2 displaying upregulated expression in IMF tissues of the L_JN pig along with downregulation of its associated miRNA (miR-339-3p). It was observed that circRNA_08840 contains the potential binding sites for miR-339 and acts as a sponge by inhibiting its expression. Overall, our results signify the potential role of circRNA_08840 in the upregulation of the TGM2 gene, whose involvement in the regulation of adipogenesis and IMF content was witnessed in various studies; moreover, it will be a good source for providing a therapeutic approach for future study.

SLC25A34 belongs to solute carrier family 25 (SLC25) member 34; being a target gene of ssc-miR-769-3p, it is well known as a mitochondrial carrier [100,101]. It has been observed that knocked-down, overexpression, or the depletion of SLC25A34 in the liver cells of mice affect the ratio of energy intermediates and lipid droplets. In addition, the alteration in the SLC25A34 gene caused altered fatty acid oxidation and glycolysis [102]. Similarly, fatty acid synthesis (FASN) is a target gene of ssc-miR-769-3p, which widely exists in mammalian cytoplasm as a key enzyme during the synthesis of fatty acid [103]. FASN was reported with higher expressions in longissimus dorsi muscles of Buksha pigs and Berkshire pigs with higher intramuscular fat content [20,22], which is in agreement with our results for both genes being upregulated in the IMF tissues of the L_JN pig and the downregulation of its associated miRNA (miR-769-3p). This concludes the role of circRNA_23437 as a sponge of miR-769-3p and its potential role in the regulation of pig intramuscular fat deposition; it also may contribute to meat quality.

Superoxide dismutase (SOD1) is a target gene of ssc-miR-503 and contributes to cellular defense against superoxide radicals (antioxidants) [104] and prevents obesity-induced changes in oxidative stress [105]. Previous studies have revealed that the oxidation of unsaturated and poly saturated fatty acids is the key process responsible for the quality deterioration of meat and meat products [26]; on the other hand, studies have emphasized the role of SOD against lipid oxidation as an antioxidant by reducing the content of MDA, which is a PUFA peroxidation product; thus, ameliorating the meat quality in pork [20]. Wen et al. (2021) observed that dietary lycopene supplementation contributed largely to improving SOD mRNA levels in the longissimus dorsi muscles of the finishing pig, which in turn improved the antioxidant capacity and meat quality [27]. Abnormal fat depositions under pathological conditions, such as primary myodystrophies, obesity, hyperglycemia, high plasma free fatty acids, and hypoxia are characterized by dysfunctional SOD1 [106]. Higher expressions in transgenic mice have been shown to reduce oxidative stress in vivo [107]. Our results also support these findings, in which the SOD1 gene is upregulated and ssc-miR-503 is downregulated, signifying circRNA_23437 as a sponge of miR-503, indicating that circRNA_23437 might also play a vital role in adipocyte differentiation and the fatty acid metabolism in pigs (Figure 9). Thus, it can be assumed that the circRNA_23437-miR-503-SOD1 axis plays a required role in lipid metabolism and meat quality by reducing oxidative damage in IMF and providing potential implications for the development of therapeutic targets to prevent insulin resistance.

Period circadian protein homolog 1 (PER1) is the target gene of miRNA-370 and plays an important role in the regulation of physiological activities and fat pathways, such as circadian rhythms, which regulate the feeding behavior-associated daily rhythms and energy metabolism [108]. In a previous study, it was revealed that the disturbance of the circadian clock function results in the dysregulation of fat metabolism, obesity, and metabolic diseases [109]. The PER1 gene, being involved in the oxidation of fatty acids and lipid biosynthesis, was observed rhythmically stimulated and suppressed by clock proteins, delivering a direct mechanism for the circadian regulation of lipid metabolic pathways [110]. It was previously revealed that obesity and type 2 diabetes significantly affect the expressions of the clock gene in subcutaneous vs. visceral adipose tissues, signifying the regulation of the expressions of the clock gene by obesity and type 2 diabetes in a tissue-specific manner [111,112,113]. This integrated study revealed that the circRNA-08840-miR370-PER1 axis may be involved in adipogenesis, fat metabolism, and the regulation of intramuscular fat tissue via circadian rhythms and circadian entrainment pathways. Therefore, circadian defects in IMF tissue can potentially lead to an altered feeding rhythm, which affects the clock gene expression, leading to metabolic syndrome. Further studies will be required to better understand the effects of circRNA-08840 on the target clock gene expression via gene editing, implicating the role in the regulation of lipid metabolism and the risk for obesity and metabolic diseases.

The collagen type V alpha 3 chain (COL5A3) gene, regulated by ssc-miR-370, is a glycoprotein and plays a crucial role in adipogenesis, the structural integrity of adipocytes, and is highly expressed in fat tissues embedded in a matrix [114]. In addition to intramuscular fat, collagen in intramuscular connective tissue (ICTM) affects meat tenderness in pigs, cattle, and goats [28,29,30]. Moreover, it is widely observed that an increased age and body weight in swine induces developmental changes in muscle composition, can be related to the increase in IMF of the carcass, and is slightly associated with tenderness and collagen. Whereas, reduced longissimus dorsi muscle IMF and collagen concentrations have been observed in pork carcasses by increasing the age under feed restrictions [31,32]. The knockdown of the COL5A3 gene significantly reduces the differentiation of the 3T3-L1 cell line of mouse preadipocytes [115]. COL5A3 knockdown has led to a reduction of dermal fat and resistance to high-fat diets in female mice. In male mice, it remarkably reduces the pancreatic islets with a subsequent increase in streptozotocin-induced apoptosis [115]. In the current study, COL5A3 gene expression was perceived as upregulated, while ssc-miR-370 was downregulated in the IMF tissues of L_JN pigs. In this study, ssc-miR-370 was disclosed as ‘sponging’ by circRNA-23437 and circRNA-08840, which may participate in the regulation of pig intramuscular fat deposition and collagen via protein digestion and the absorption pathways, contributing to meat quality. Hence, it can be assumed that the significance of extracellular matrix (ECM) in cell environments may influence the proper development and functioning of specialized cells, such as adipocytes, IMF, and skeletal muscles.

## 5. Conclusions

In the current study, the differences in the expressions of IMF-associated genes in two pig breeds (Large White and Laiwu pigs) were examined in IMF tissues of longissimus dorsi muscles regarding fat accumulation tendencies, which indicated that circRNA and miRNA expressions might be responsible for variations in adipogenesis in different pig breeds. circRNA_23437 and circRNA_08840 have binding sites for miR_370, miR-769-3p, miR-339-3p, and miR-503, and act as sponges and facilitate the regulation of fat deposition and lipid metabolism via the TFG-beta signaling/signaling pathways regulating the pluripotency of stem cells/hippo signaling pathways/AMP signaling pathways/adipocytokine signaling pathways. However, further investigations are required to explore the profound effects of circRNA_23437 and circRNA_08840 on the IMF biology of pigs and the roles of SOD1, SLC5A6, COL5A3, TGM2, SLC25A34, FASN, and PER1 in fat deposition, meat quality, and as biomarkers of abnormal fat metabolism using advance gene editing techniques. Their evolutionary trajectories, expression profiling, functional enrichments, subcellular localizations, and structural advances, with high-throughput protein modeling following genomic organizations, will provide new insights into underlying molecular mechanisms, and a much-needed qualitative framework for future research to improve meat quality and therapeutic approaches to treat lipid metabolic syndromes.

## Figures and Tables

**Table 1:** Differentially expressed miRNAs in the IMF tissues of Large White and Laiwu pigs.

miR_Name	Mean (D)	Mean (L)	Log2 (Fold Change) (L/D)	*p*_Value	q_Value
ssc-miR-370	152.6247008	28.35987849	−2.42807	8.04 × 10^−78^	1.10 × 10^−76^
ssc-miR-127	3416.29341	954.4726318	−1.83966	0	0
ssc-miR-339-3p	4.933988172	1.627206143	−1.60036	0.011932	4.32 × 10^−2^
ssc-miR-339	483.5308409	7.438656653	−6.02242	0	0
ssc-miR-7137-3p	6.907583441	2.789496245	−1.30818	0.010358	3.83 × 10^−2^
ssc-miR-503	19.73595269	7.438656653	−1.40771	4.39 × 10^06^	2.37 × 10^−5^
ssc-miR-769-3p	40.12977047	17.89926757	−1.16477	1.67 × 10^08^	1.05 × 10^−7^

**Table 2:** circRNA-miRNA interactions and their expressions in the IMF tissues of Large White and Laiwu pigs. Note: the position represents the start site where the miRNA binds to the circRNA region. (Large White is a reference group).

miRNA	miRNA Expression	Transcript	circRNA Expression	Total Score	Total Energy	Max Score	Max Energy	miRNA Length	Transcript Length	Position
ssc-miR-127	Down	circRNA_08840 (chr13:217685619_217779051_-)	Up	158	−31.47	158	−31.47	22	93,433	7134
ssc-miR-339-3p	Down	circRNA_08840 (chr13:217685619_217779051_-)	Up	152	−32.13	152	−32.13	21	93,433	36837
ssc-miR-339	Down	circRNA_08840 (chr13:217685619_217779051_-)	Up	309	−60.47	156	−30.36	21	93,433	63989 48747
ssc-miR-370	Down	circRNA_08840 (chr13:217685619_217779051_-)	Up	476	−105.8	166	−40.11	22	93,433	10849 46,872 69308
ssc-miR-7137-3p	Down	circRNA_08840 (chr13:217685619_217779051_-)	Up	336	−68.04	171	−36.35	22	93,433	13332 48860
ssc-miR-503	Down	circRNA_23437 (chr7:24626858_24686216_-)	Up	176	−32.35	176	−32.35	23	57,856	14200
ssc-miR-769-3p	Down	circRNA_23437 (chr7:24626858_24686216_-)	Up	176	−36.55	176	−36.55	23	57,856	45734
ssc-miR-370	Down	circRNA_23437 (chr7:24626858_24686216_-)	Up	166	−32.79	166	−32.79	22	57,856	39180

## Data Availability

The materials and datasets used and analyzed during the present study are available from the corresponding author upon reasonable request.

## Supplementary Materials

The following supporting information can be downloaded at: https://www.mdpi.com/article/10.3390/cells11172638/s1, Table S1: list of all Primers used in current study.
